# Characteristic of Tumor Regrowth After Gamma Knife Radiosurgery and Outcomes of Repeat Gamma Knife Radiosurgery in Nonfunctioning Pituitary Adenomas

**DOI:** 10.3389/fonc.2021.627428

**Published:** 2021-03-05

**Authors:** Yanli Li, Lisha Wu, Tingting Quan, Junyi Fu, Linhui Cao, Xi Li, Shunyao Liang, Minyi Huang, Yinhui Deng, Jinxiu Yu

**Affiliations:** ^1^ Department of Endocrinology, The Second Affiliated Hospital of Guangzhou Medical University, Guangzhou, China; ^2^ Department of Medical Oncology, Sun Yat-sen Memorial Hospital, Sun Yat-sen University, Guangzhou, China; ^3^ Department of Radiology, Sun Yat-sen University Cancer Center, State Key Laboratory of Oncology in South China, Collaborative Innovation Center for Cancer Medicine, Guangzhou, China; ^4^ Department of Neurology, The Second Affiliated Hospital of Guangzhou Medical University, Guangzhou, China; ^5^ Department of Traditional Chinese Medicine, Sun Yat-sen Memorial Hospital, Sun Yat-sen University, Guangzhou, China; ^6^ Department of Radiology, The Second Affiliated Hospital of Guangzhou Medical University, Guangzhou, China; ^7^ Department of Radiotherapy, The Second Affiliated Hospital of Guangzhou Medical University, Guangzhou, China

**Keywords:** gamma knife, radiosurgery, regrowth, pituitary adenoma, aggressive, nonfunctioning

## Abstract

**Objective:**

This study aimed to report the characteristic of tumor regrowth after gamma knife radiosurgery (GKRS) and outcomes of repeat GKRS in nonfunctioning pituitary adenomas (NFPAs).

**Design and Methods:**

This retrospective study consisted of 369 NFPA patients treated with GKRS. The median age was 45.2 (range, 7.2–84.0) years. The median tumor volume was 3.5 (range, 0.1–44.3) cm^3^.

**Results:**

Twenty-four patients (6.5%) were confirmed as regrowth after GKRS. The regrowth-free survivals were 100%, 98%, 97%, 86% and 77% at 1, 3, 5, 10 and 15 year, respectively. In multivariate analysis, parasellar invasion and margin dose (<12 Gy) were associated with tumor regrowth (hazard ratio [HR] = 3.125, 95% confidence interval [CI] = 1.318–7.410, p = 0.010 and HR = 3.359, 95% CI = 1.347–8.379, p = 0.009, respectively). The median time of regrowth was 86.1 (range, 23.2–236.0) months. Previous surgery was associated with tumor regrowth out of field (p = 0.033). Twelve patients underwent repeat GKRS, including regrowth in (n = 8) and out of field (n = 4). Tumor shrunk in seven patients (58.3%), remained stable in one (8.3%) and regrowth in four (33.3%) with a median repeat GKRS margin dose of 12 (range, 10.0–14.0) Gy. The actuarial tumor control rates were 100%, 90%, 90%, 68%, and 68% at 1, 3, 5, 10, and 15 years after repeat GKRS, respectively.

**Conclusions:**

Parasellar invasion and tumor margin dose (<12 Gy) were independent risk factors for tumor regrowth after GKRS. Repeat GKRS might be effective on tumor control for selected patients. For regrowth in field due to relatively insufficient radiation dose, repeat GKRS might offer satisfactory tumor control. For regrowth out of field, preventing regrowth out of field was the key management. Sufficient target coverage and close follow-up might be helpful.

## Introduction

Nonfunctioning pituitary adenomas (NFPAs) represent about 30% (1) of pituitary tumors. The managements of NFPAs include surgical resection, radiotherapy, medical treatment, and observation. Surgical resection is firstly recommended as the primary treatment of symptomatic patients with NFPA (2). Radiotherapy is recommended for residual or recurrent NFPAs (3). When patients are not candidate to surgical resection because of significant comorbidities, an advanced age or cavernous sinus invasion, radiotherapy may be used as primary management (4). Gamma Knife radiosurgery (GKRS) which has advantages of a highly precise, better dose conformity and focused delivery of radiation in a single session, is one of the best radiation technique and essential part in the treatment of pituitary tumors. As previous publications reported (4–12), GKRS has been proved to offer a high tumor control rate of 83–95% and a low new-onset hypopituitarism rate of 9–32% for pituitary adenomas. Treatment failure after GKRS for NFPAs consists of progressive cystic enlargement, tumor apoplexy and tumor regrowth (4). Tumor regrowth is the most common type of treatment failure in GKRS for NFPAs. Most publications reported tumor recurrence in 0–9.6% of treated patients with NFPA after GKRS (11, 13–18). However, there are few studies reporting the characteristics of tumor regrowth after GKRS and outcomes of repeat GKRS. Since 1993, the Second Affiliated Hospital of Guangzhou Medical University has more than 26 years’ experience in using Gamma Knife (Elekta, Stockholm, Sweden) for NFPAs. To report the characteristics of tumor regrowth and outcomes of repeat GKRS for NFPA patients with tumor regrowth after GKRS, we performed a single-center study.

## Methods

### Patient Population

Between 1993 and 2016, there were 2557 patients with pituitary adenomas treated with GKRS at the Second Affiliated Hospital of Guangzhou Medical University. Most of patients were lost to follow up because of coming from a long distance. Finally, there were only 751 pituitary adenoma patients had clinical and sufficient follow-up (>12 months) information at our hospital. Of the 751 patients, 369 NFPA patients were enrolled in this study. The patients were diagnosed by surgical pathology or MRI findings. There was no evidence of hormonal hypersecretion in these patients. This retrospective study was approved by the institutional committee of the Second Affiliated Hospital of Guangzhou Medical University.

### Clinical and Radiological Evaluations

All of patients were routinely followed up with MRI of the sellar and clinical evaluations. No matter when it was possible, patients took follow-up examination at our hospital. If not, clinical information, MRI and laboratory tests were sent and reviewed at our center. The follow-up evaluations were collected and reviewed by the treating radiologists and clinicians.

**Figure 1:**
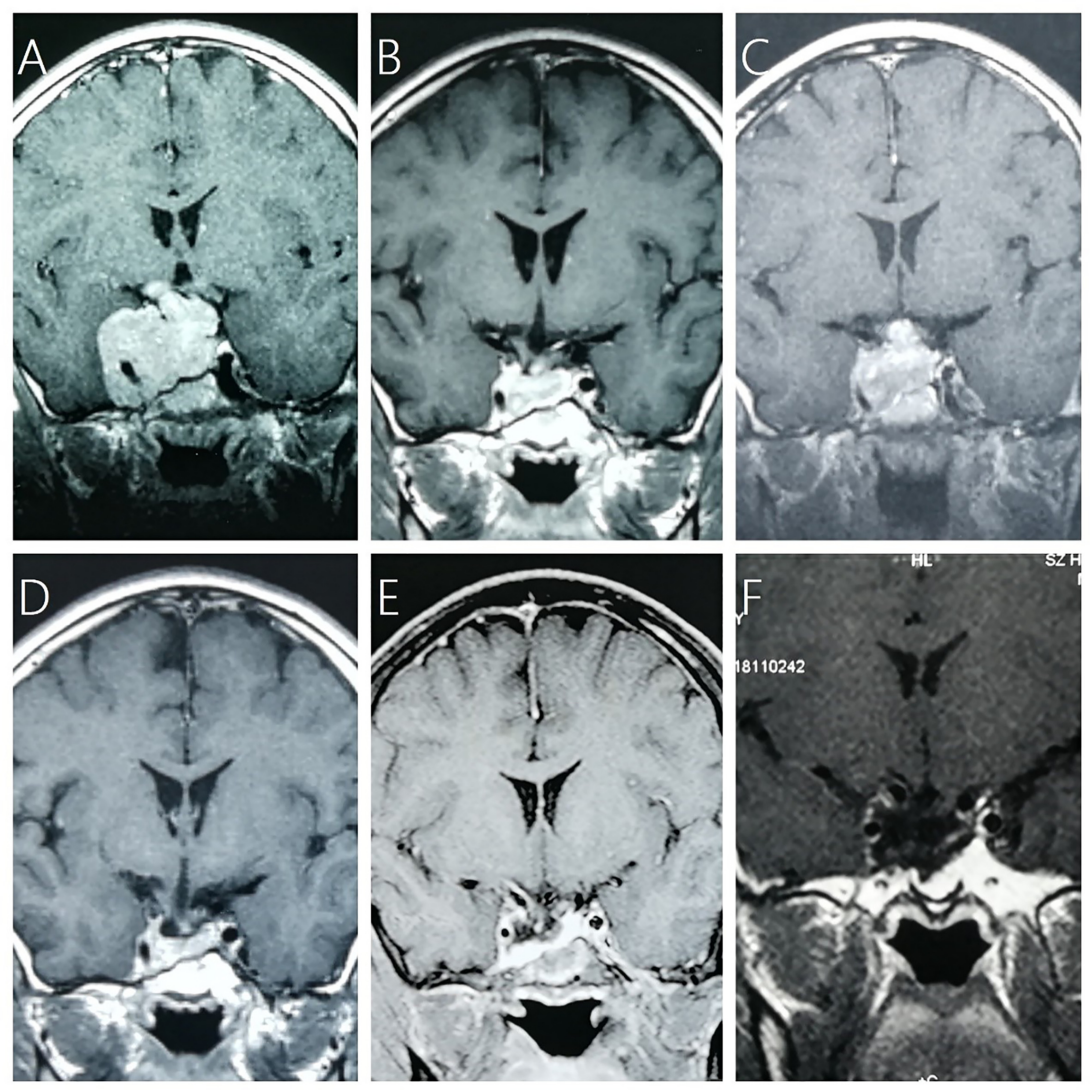
A 13-year-old boy with NFPA (max diameter of 7.6 cm) received adjuvant GKRS (10 Gy/35%) after subtotal resection and repeat GKRS (12 Gy/35%) for tumor regrowth at 36.5 months after prior GKRS. **(A)** contrast-enhanced coronal T1-weighted magnetic resonance imaging (MRI) scans showed residual giant NFPA after surgical resection. **(B)** MRI showed tumor shrinkage at 24.6 months after GKRS. **(C)** MRI showed tumor regrowth at 37.9 months after prior GKRS. **(D)** MRI showed tumor shrinkage at 10.1 months after repeat GKRS. **(E)** MRI showed tumor shrinkage at 68.8 months after repeat GKRS. **(F)** MRI showed tumor shrinkage at 205.0 months after repeat GKRS.

**Figure 2:**
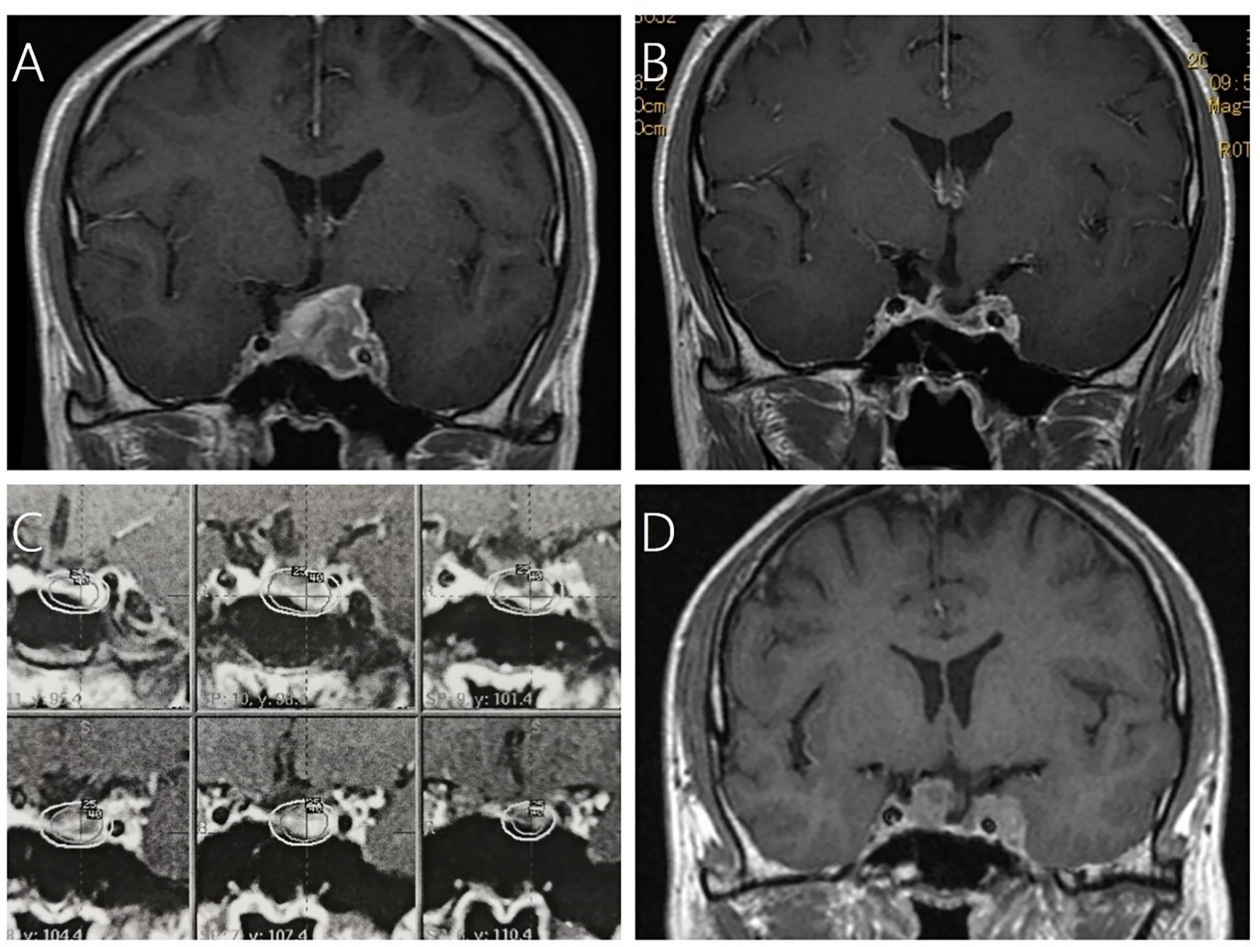
A 43-year-old male patient with residual NFPA after surgical resection received GKRS and developed tumor regrowth out of field at 71 months after GKRS. **(A)** contrast-enhanced coronal T1-weighted magnetic resonance imaging (MRI) scans showed pituitary adenoma. **(B)** MRI showed subtotal resection for pituitary adenoma after 3.5 months. **(C)** Dose distribution of adjuvant GKRS after surgical resection. **(D)** MRI showed tumor regrowth was either in the sellar as well as in the cavernous sinus out of field.

Tumor dimensions were got from MR imaging by manual. The tumor dimensional indices were measured and recorded in three orthogonal planes: transverse (TR), anteroposterior (AP), and craniocaudal (CC). The tumor volumes were estimated using the formula: V = (π × [TR × AP × CC])/6 (19). Considering the irregular shape of some tumors, tumor volume measurement was only a rough estimate of the actual volume. Tumor progression was defined as tumor enlargement at least 20% in tumor volume. Tumor shrinkage was defined as at least a 20% shrinkage in tumor volume. Stable tumor was defined as tumor volume change within 20%. Tumor regrowth was defined as new lesion detected on follow-up MRI or regrowth on residual tumor. Tumor regrowth on adjacent or within the prescribed isodose was considered as regrowth in field (**Figure 1**). Tumor regrowth outside the prescribed isodose was considered as regrowth out of field (**Figure 2**). The Knosp grade 3 or 4 was considered as parasellar invasion. The tumor close to optic structure (<2 mm) was considered as suprasellar extension.

### Gamma Knife Radiosurgery Technique

The procedure was performed using Leksell Gamma Knife. Model B Leksell Gamma Knife Unit was used until April 2014 and was then replaced by Perfexion Unit (Elekta Instrument, Inc.). Stereotactic Leksell frame placement was performed under local anesthetic. Following frame placement, thin-slice stereotactic MR imaging with the administration of intravenous contrast material was performed through the sellar. The maximal dose to the optic pathway was ≤10 Gy. Small collimators of 4 and 8 mm were mainly used to get better conformality.

### Statistical Analysis

The normal distribution of continuous variables was checked by Kolmogorov–Smirnov test. The mean ( ± SEM) was used to describe continuous variables with normal distribution. The median and interquartile ranges (IQR) was used to describe variables not normally distributed. F test was used for homogeneity of variance in continuous variables. Independent-sample t test was used to compare means of continuous variables with normal distribution. When continuous variables were not normally distributed, Wilcoxon rank sum test was used. Chi-square test and Fisher exact test were used for statistical analysis of categorical variables. Log-rank test statistics and a step forward likelihood ratio method of Cox proportional hazard models were used for univariate and multivariate analysis, respectively. Kaplan-Meier curves were plotted for regrowth-free survival. Probability values < 0.05 were defined as statistically significant. For statistical analysis, IBM’s SPSS (version 26.0) was used.

## Results

### Patient Characteristics

There were 369 NFPA patients in this study. The population consisted of 185 male (50.1%) and 184 female (49.9%) patients with a median age of 45.2 (range, 7.2–84.0) years. The median follow-up was 60.8 (range, 12.8–283.0) months. The median tumor volume was 3.5 (range, 0.1–44.3) cm^3^. There were four patients (1.1%) underwent radiation before GKRS. There were 173 patients (46.9%) treated with adjuvant GKRS after surgery. There were 162 patients (43.9%) with suprasellar extension and 138 patients (34.8%) with parasellar invasion. The median tumor margin dose was 13.3 (range, 8.0–22.0) Gy at a median prescription isodose 40% (range, 25–71%). The median maximum dose was 33.3 (range, 14.0–66.7) Gy (**Table 1**).

**Table 1:** Baseline clinical characteristics of 369 patients with nonfunctioning pituitary adenomas and GKRS parameters.

Characteristic	value
Male/Female, n (%)	185/184 (50.1/49.9)
Median age, (range), years	45.2 (7.2–84.0)
Median FU length, (range), months	60.8 (12.8–283.0)
Median tumor volume at GKRS, (range), cm^3^	3.5 (0.1–44.3)
Previous radiotherapy, n (%)	4 (1.1)
Prior surgical resection, n (%)	173 (46.9)
Parasellar invasion, n (%)	138 (34.8)
Suprasellar extension, n (%)	162 (43.9)
GKRS parameters	
Median tumor margin radiation dose, (range), Gy	13.3 (8.0–22.0)
Median maximum radiation dose, (range), Gy	33.0 (14.0–66.7)
Median prescription isodose, (range), %	40.0 (25.0–71.0)

FU, follow up; GKRS, gamma knife radiosurgery.

### Risk Factors Associated With Tumor Regrowth

**Figure 3:**
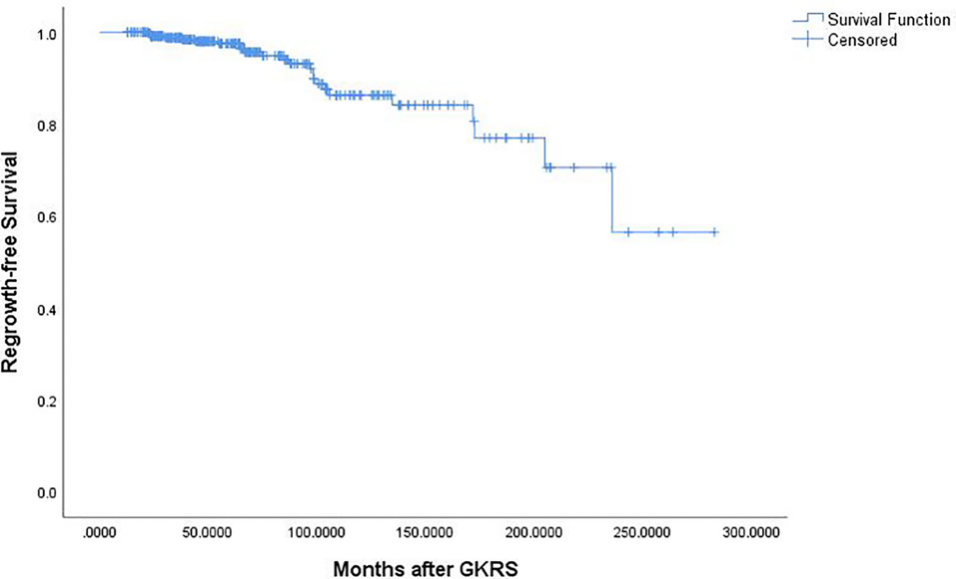
Kaplan–Meier curve of tumor regrowth-free survival.

**Figure 4:**
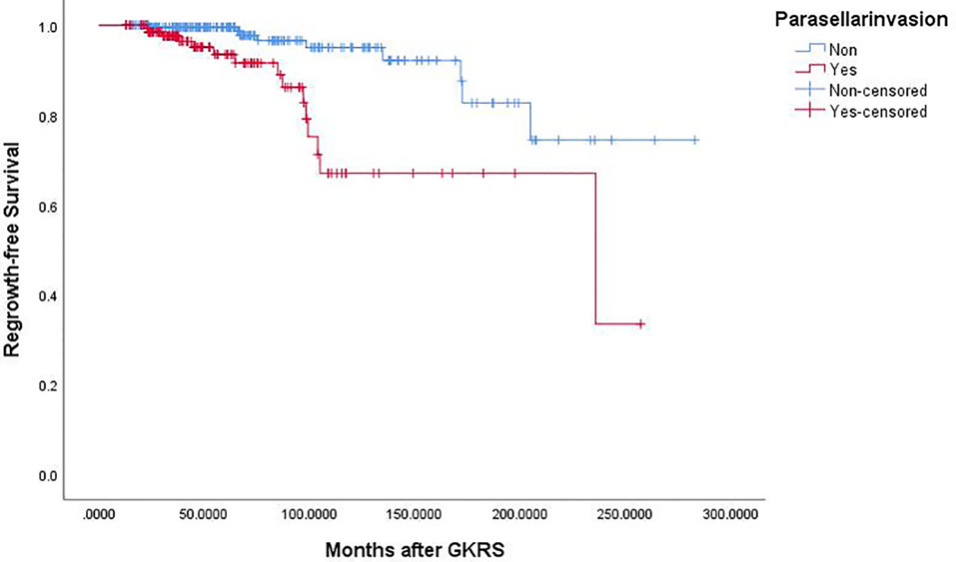
Kaplan–Meier curve of tumor regrowth-free survival of parasellar invasion (p = 0.000).

**Figure 5:**
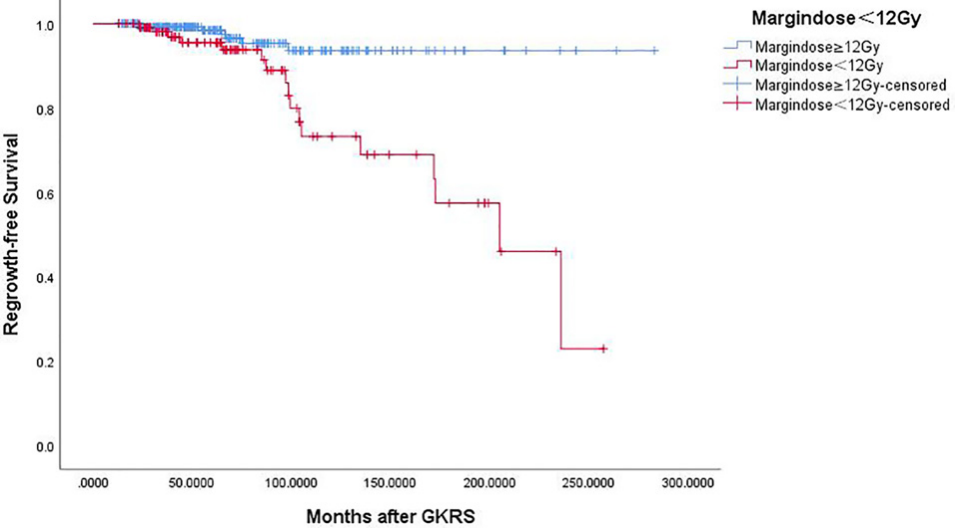
Kaplan–Meier curve of tumor regrowth-free survival of tumor margin dose ≥12 Gy vs. <12 Gy. Tumor margin dose <12 Gy showed a lower tumor regrowth-free survival rate (p = 0.000).

Of the 369 NFPA patients who underwent GKRS, 24 patients (6.5%) confirmed as tumor regrowth. The regrowth-free survivals were 100%, 98%, 97%, 86% and 77% at 1, 3, 5, 10, and 15 year, respectively (**Figure 3**). In univariate analysis, risk factors associated with tumor regrowth included prior surgical resection (p = 0.034), parasellar invasion (p ≤ 0.001) (**Figure 4**), tumor margin dose (<12 Gy) (p ≤ 0.001) (**Figure 5**), and tumor volume (≥5 cm^3^) (p = 0.003). In multivariate analysis, only parasellar invasion and tumor margin dose (<12 Gy) were significantly related with tumor regrowth (hazard ratio [HR] = 3.125, 95% confidence interval [CI] = 1.318–7.410, p = 0.010 and HR = 3.359, 95% CI = 1.347–8.379, P = 0.009, respectively) (**Table 2**).

**Table 2:** Results of univariate and multivariate analyses for tumor regrowth after GKRS.

Variables	Tumor regrowth			
	Univariate, *p*	Multivariate, *p*	HR	95% CI
Age (≥55 years)	0.847	NA	NA	NA
Sex (male VS female)	0.649	NA	NA	NA
Prior surgical resection	0.034^*^	0.169	NA	NA
Parasellar invasion	≤0.001^*^	0.010^*^	3.125	1.318–7.410
Suprasellar invasion	0.096	0.194	NA	NA
Tumor margin dose (<12 Gy)	≤0.001^*^	0.009^*^	3.359	1.347–8.379
Tumor volume (≥5 cm^3^)	0.003^*^	0.920	NA	NA

GKRS, gamma knife radiosurgery; CI, confidential interval; HR, hazards ratio; NA, not available.

^*^Statistically significant (P < 0.05).

### Characteristics of Tumor Regrowth

Of the 24 patients with tumor regrowth after GKRS, there were 13 male (54.2%) and 11 female (45.8%) patients with a mean age of 40.7 (median, 49.2, range, 16.4–70.2) years. The mean follow-up was 144.1 (median, 134.1, range, 22.5–260.8) months. There were 15 (62.5%) and 9 patients (37.5%) with tumor regrowth in and out of field, respectively. There were 14 patients (58.3%) underwent surgical resection previously. The mean tumor volume at prior GKRS was 13.1 (median, 9.8, range, 0.9–34.8) cm^3^. There were 22 patients (91.7%) with suprasellar extension and 17 patients (70.8%) with parasellar invasion. The mean prior GKRS margin dose was 10.0 (median, 10.0, range, 9.0–17.0) Gy. The mean prior GKRS maximum dose was 33.1 (median, 33.2, range, 25.0–40.0) Gy. The mean time of regrowth was 91.8 (median, 86.1, range, 23.2–236.0) months. The characteristics of the 24 patients, grouped according to the type of tumor regrowth, were summarized in **Table 3**. There were more patients who previously underwent surgery developed tumor regrowth out of field (p = 0.033). the proportion of gender, parasellar invasion, suprasellar extension, age, margin dose, maximum dose, tumor volume, and time of regrowth, were similar in the two groups (**Table 3**).

**Table 3:** Characteristics of 24 NFPA patients grouped according to the type of tumor regrowth after GKRS.

Characteristic	Regrowth in field (n = 15)	Regrowth out of field (n = 9)	All patients (n = 24)	P value
Female sex, n (%)	7 (46.7)	4 (44.4)	11 (45.8)	1.000
Mean age at prior GKRS (years)	41.1 ± 3.8	39.9 ± 3.9	40.7 ± 2.7	0.833
Parasellar invasion, n (%)	9 (60)	8 (88.9)	17 (70.8)	0.191
Suprasellar extension, n (%)	13 (86.7)	9 (100)	22 (91.7)	0.511
Previous surgical resection, n (%)	6 (40)	8 (88.9)	14 (58.3)	0.033^*^
Prior GKRS margin dose, median (IQR), (Gy)	10.0 (9.0–12.0)	11.0 (10.0–12.8)	10.0 (9.9–12.6)	0.528
Mean prior GKRS maximum dose (Gy)	33.3 ± 1.0	33.4 ± 0.6	33.1 ± 0.6	0.723
Time of regrowth, median (IQR), (months)	85.0 (64.8–134.8)	97.0 (23.5–102.2)	86.1 (46.8–104.8)	0.325
Mean tumor volume at prior GKRS, median (IQR), (cm^3^)	9.4 (5.8–15.7)	13.6 (4.6–27.9)	9.8 (5.3–20.1)	0.421

Data are expressed as number, mean ± SEM, median and IQR, or percentage.

GKRS, gamma knife radiosurgery; IQR interquartile range.

^*^Statistically significant (P < 0.05).

### Further Treatment and Outcomes of Repeat GKRS

Among the 24 patients, 16 patients (66.7%) underwent repeat GKRS alone, 2 patients (8.3%) underwent surgery, 2 patients (8.3%) underwent surgery and repeat GKRS, 2 patients (8.3%) were under observation, 2 patients were lost to follow up. There was no other medical treatment except hormone supplement in these patients.

**Figure 6:**
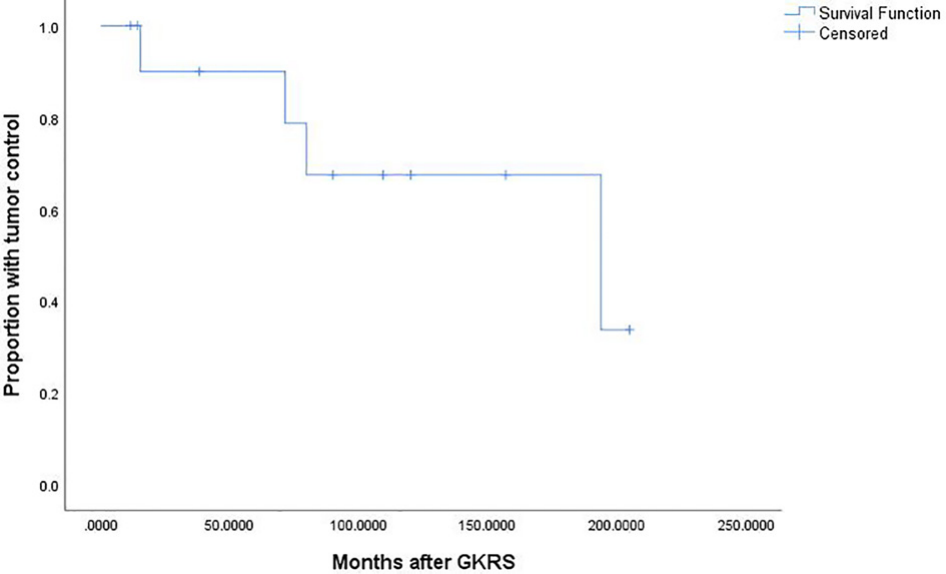
Kaplan-Meier curve of tumor control for the 12 patients who undergoing repeat GKRS. The actuarial tumor control rates were 100%, 90%, 90%, 68%, and 68% at 1, 3, 5, 10, and 15 years after repeat GKRS, respectively.

There were 18 patients underwent repeat GKRS. Six patients were lost to follow up. Finally, only 12 patients underwent repeat GKRS alone had follow-up MRI. The data was showed in **Table 4**. The patient population consisted of six male (50%) and 6 females (50%) patients with a median age of 46.7 (range, 16.4–70.2) years. There were 8 (66.7%) and 4 patients (33.3%) with tumor regrowth in and out of field, respectively. There were 8 patients (66.7%) with parasellar invasion. The median previous GKRS margin dose was 10.0 (range, 9.0–15.5) Gy. The median previous GKRS maximal dose was 33.2 (range, 25.0–36.0) Gy. The median tumor volume at repeat GKRS was 9.8 (range, 0.6–66.8) cm^3^. The median repeat margin dose and maximum dose was 12 (range, 10.0–14.0) Gy and 33.2 (range, 28–40) Gy, respectively. Finally, with a median imaging follow-up of 84.8 (range,11.4–205.0) months after repeat GKRS, tumor shrunk in 7 patients (58.3%), remained stable in 1 patient (8.3%) and tumor regrowth in 4 patients (33.3%). The actuarial tumor control rates were 100%, 90%, 90%, 68%, and 68% at 1, 3, 5, 10, and 15 years after repeat GKRS, respectively (**Figure 6**). Among the eight patients with tumor control, there were two patients with short imaging follow-up of 11.4 and 14.1 months, respectively, which might overestimate tumor control rate in this study. For the patient with tumor shrinkage at imaging follow-up of 11.4 months, we had follow-up by telephone at 216.9 months after repeat GKRS, the patient had a good quality of life and nothing to complain, including headache, visual impairment, and cranial nerve impairment. Another patient with a large tumor volume of 66.8 cm^3^ remained stable at 14.1 months was lost to follow-up. Among the four patients with tumor regrowth after repeat GKRS, three patients presented with tumor regrowth out of field. In these three patients, all of the tumors within the repeat GKRS radiation field were shrinkage, one patient received 3^rd^ GKRS for tumor regrowth in cavernous sinus, one patient was under observation, and another patient was lost to follow-up. It was indicated that these patients were heavily infiltrating NFPA and should be cautious of tumor regrowth out of field. Another patient with tumor regrowth in field previously presented tumor regrowth in field again after repeat GKRS and was advised to receive surgical resection. It was indicated that the tumor might be resistant to radiation. After repeat GKRS, one patient who presented with tumor regrowth in the cavernous sinus occurred new oculomotor neuropathy, another patient whose optic chiasm was compressed by tumor regrowth in the suprasellar region occurred new or worsened visual impairment.

**Table 4:** Imaging outcomes of repeat GKRS for 12 NFPA patients with tumor regrowth after GKRS.

Sex/Age	Type of regrowth	Previous GKRS dose	Repeat GKRS dose	Imaging outcome	FU after repeat GKRS (months)
Male/31.4	Out of field	10.0 Gy at 30%	14.4 Gy at 40%	Regrowth (out of field)	71.4
Female/49.1	In field	15.1 Gy at 50%	10.0 Gy at 25%	Shrinkage	11.4
Female/55.9	In field	9.0 Gy at 36%	11.7 Gy at 35%	Regrowth (out of field)	193.8
Female/48.1	In field	10.0 Gy at 30%	13.0 Gy at 40%	Shrinkage	89.9
Male/16.4	In field	10.0 Gy at 35%	12.0 Gy at 40%	Shrinkage	205.0
Male/53.6	In field	11.6 Gy at 35%	11.7 Gy at 35%	Shrinkage	156.9
Female/70.2	In field	10.0 Gy at 30%	14.0 Gy at 50%	Shrinkage	38.2
Female/31.9	In field	9.9 Gy at 30%	11.8 Gy at 33%	Shrinkage	109.3
Female/49.7	Out of field	10.0 Gy at 30%	10.5 Gy at 35%	Stable	14.1
Male/43.1	Out of field	11.0 Gy at 35%	12.0 Gy at 40%	Regrowth (out of field)	15.3
Male/22.3	In field	12.0 Gy at 35%	13.2 Gy at 40%	Regrowth (in field)	79.6
Male/45.4	Out of field	14.4 Gy at 40%	14.0 Gy at 40%	Shrinkage	120.0

FU, follow up; GKRS, gamma knife radiosurgery; TV, tumor volume.

Risk factors such as age, sex, parasellar invasion, suprasellar extension, prior surgery, repeat GKRS margin dose, maximal repeat GKRS radiation dose, type of regrowth, and time of regrowth were analyzed. No factors were significantly associated with progression. The small number of cases limited statistical power.

## Discussion

Our study was a single-center series reporting the characteristic of tumor regrowth after GKRS and outcomes of repeat GKRS in NFPA patients. In the current study, 24 patients (6.5%) were confirmed as regrowth after GKRS. The regrowth-free survivals were 100%, 98%, 97%, 86% and 77% at 1, 3, 5, 10, and 15 years, respectively. The median time of regrowth was 86.1 (range, 23.2–236.0) months. In multivariate analysis, only parasellar invasion and tumor margin dose (<12 Gy) were significantly associated with tumor regrowth. Twelve patients underwent repeat GKRS, including regrowth in (n = 8) and out of field (n = 4). Tumor shrunk in seven patients (58.3%), remained stable in one (8.3%) and regrowth in four (33.3%) with a median repeat GKRS margin dose of 12 (range, 10.0–14.0) Gy. The actuarial tumor control rates were 100%, 90%, 90%, 68%, and 68% at 1, 3, 5, 10 and 15 years after repeat GKRS, respectively.

In previous studies, 0–9.6% of NFPA patients occurred tumor recurrence after GKRS (11, 13–18). In a large study of 543 patients with pituitary adenomas by Losa et al. (18), there were more patients with NFPA than functioning pituitary adenomas had a tumor recurrence (9.6% VS 4.8%). In the 272 NFPA patients, there were 26 patients (9.6%) developed tumor recurrence, which was higher than our study. In the NFPA group, there were no risk factors associated with tumor recurrence. Sheehan et al. (9) reported a pooled analysis of data from nine center in North America, 31 of 469 NFPA patients (6.6%) developed tumor regrowth after a shorter median follow-up of 36 months. Tumor volume at GKRS was the only risk factor associated with tumor recurrence. Sun et al. (5) also reported parasellar invasion was risk factor associated with tumor control in the treatment of GKRS for postsurgical NFPAs. Radiosurgery with single doses of ≥12 Gy is recommended for greater local tumor control rate of ≥90% in a systematic review and evidence-based guideline (3). In our study, a large tumor volume and tumor close to optic nerve were the reasons of a relative low tumor margin dose to tumor. Therefore, these patients were prone to regrow due to a relative low dose. For the tumors with large volume or close to optic nerve, multisession GKRS or fractioned stereotactic radiation therapy might have advantage for tumor control comparing with single session GKRS. Tomotherapy, Cyberknife or linear accelerator were not available in our hospital. Leksell Gamma Knife Unit B was replaced by Perfexion Unit until 2014. These may be disadvantage for treatment of tumors with large volume.

In current study, with a median imaging follow-up of 84.8 (range,11.4–205.0) months after repeat GKRS for 12 patients with regrowth, tumor shrunk in seven patients (58.3%), remained stable in one patient (8.3%), and tumor regrowth in four patients (33.3%). The actuarial tumor control rates were 100%, 90%, 90%, 68%, and 68% at 1, 3, 5, 10, and 15 years after repeat GKRS, respectively. In the study of Losa et al. (18), 16 of 26 NFPA patients received GKRS as further therapy, and 15 of them had final outcomes. With median follow-up of 68 (range, 14–167) months in these patients, tumor improving in 1 patient (6.7%), remained stable in 13 patients (86.7%), only 1 patient (6.7%) with tumor progression. The actuarial tumor control rates were 93 and 93% at 5 and 10 years, respectively, which were higher than our study. However, the proportion of tumor improving was much lower than our study. Besides, the only patient with tumor improving received GKRS and temozolomide. Did the tumor shrinkage was due to GKRS or temozolomide or both of them? What’s more, the definition of tumor improving and stable were not available in the literature.

In this study, we defined two clearly distinct patterns of tumor regrowth after GKRS: tumor regrowth in and out of previous radiation field. The tumor regrowth in field was more frequent than out of field (66.7 VS 33.3%) in our study. In the study of Losa et al. (18), of the 26 patients, there were 18 patients (69.2%) developed recurrence out of field, which was higher than our study. We found previous surgery was significantly associated with tumor regrowth out of field (p = 0.033). In the study of Losa et al. (18), 91.5% of NFPA patients had previous surgery. However, because of significant comorbidities, an advanced age, preoperative functional status and cavernous sinus invasion, only 14 patients (58.3%) had previous surgery in our study. A higher proportion of surgery contributed to a higher proportion of recurrence out of field. The underlying pathogenesis might be different in the two kind of tumor regrowth, which might have influence on prognostic and therapeutic outcomes. Tumor regrowth out of field usually represented insufficient target coverage because of the tumor infiltrating into surrounding structures or difficulty of differentiating postsurgical changes from residual tumor. Therefore, the tumor target contouring should be performed on presurgical and postsurgical MRI, in order to avoid missing the small residual tumor. Thus, the tumor regrowth out of field seemed to be a low correlation with radiation resistance. In the four patients presented tumor regrowth after repeat GKRS, three of them who were regrowth out of field still showed well response to repeat GKRS radiation field. In the study of Losa et al. (18), most patients with “out of field” recurrence also responded well to GKRS and had stable disease at last follow-up. Preventing tumor regrowth out of field was the key management. Sufficient target coverage and close MRI follow-up might be helpful. Nevertheless, the reasons of tumor regrowth in field might consist of radiation resistance and relatively insufficient radiation dose. If the tumors were resistant to radiation, it might present with aggressive behavior and limit the treatment options. If the tumors regrowth in field were due to relatively insufficient radiation dose, then a high radiation dose might be helpful to control tumor regrowth. In the eight patients with tumor regrowth in field in our study, seven patients received a higher repeat GKRS margin dose than previous dose, two patients (25%) developed regrowth again, including regrowth in field (n = 1) and out of field (n = 1). The patient regrowth in field after repeat GKRS should be considered more resistant to radiation than other patients.

The 2018 European Society of Endocrinology Clinical Practice Guidelines for the management of aggressive pituitary tumors and carcinomas suggested aggressive pituitary adenomas should be considered in patients with a radiologically invasive tumor and unusually rapid tumor growth rate, or clinically relevant tumor growth despite optimal standard therapies (surgery, radiotherapy and conventional medical treatments) (20). Of the 12 patients receiving repeat GKRS in our study, only 8 patients (66.7%) were radiologically cavernous sinus invasion. Another patient without cavernous sinus invasion was considered refractory to radiation. Most of cases had characteristics of aggressive behavior. There was no standard therapy for aggressive pituitary adenomas. There had been a low-level evidence for temozolomide in small case series (21–26). Recently, an international survey of clinical practice (27) recommended temozolomide as first line chemotherapeutic treatment of aggressive pituitary tumors or pituitary carcinomas. As previous reported, 69% (28) of patients could obtain complete response, partial response or stable disease. The success rate of clear tumor volume reduction (complete response or partial response) was 42% (28). There was rare data about radiotherapy for aggressive pituitary adenomas. In the international survey of clinical practice (27), there were 10 patients underwent radiotherapy as second and third line treatments. Six patients (60%) developed progression. The effect was limited. Our study reported a better tumor control rate. Perhaps, it was due to the heterogenous of the tumors between the studies. For aggressive pituitary tumors, patients should be treated by multidisciplinary team consisting of a neurosurgeon, radiation oncologist, radiologist, endocrinologist and pathologist.

In this study, there were several limitations should be noticed. Firstly, this was a single-center retrospective study with small sample size and thereby reflected selection and treatment biases, as well as limiting statistical power. Secondly, some patients did not receive surgical resection before GKRS, the pathological information was not available, which might indicate aggressive behavior in these patients. Thirdly, tumor volume measurement in this study was only a rough estimate of the actual volume because of the irregular shape of some pituitary tumors. Fourthly, because many patients came from a long distance from nationwide, endocrine tests were usually took in local hospital for their convenience. Therefore, endocrine evaluations before and after GKRS were incomplete in this study.

In this study, parasellar invasion and tumor margin dose (<12 Gy) were independent risk factors for tumor regrowth after GKRS. Tumor regrowth may occur several years after GKRS, long-term regular follow-up is necessary. Tumor regrowth in and out of field may possess different mechanisms and affect prognosis. Repeat GKRS might be effective on tumor control for selected patients. For the pattern of regrowth in field due to relatively insufficient radiation dose, repeat GKRS may still offer satisfactory tumor control rate. For tumor regrowth out of field, preventing tumor regrowth out of field was the key management. Sufficient target coverage and close MRI follow-up might be helpful. All in all, for better management of aggressive pituitary tumors, it should be conducted by a multidisciplinary team consisting of a neurosurgeon, radiation oncologist, radiologist, endocrinologist and pathologist.

## Data Availability Statement

The original contributions presented in the study are included in the article/supplementary material. Further inquiries can be directed to the corresponding author.

## Ethics Statement

The studies involving human participants were reviewed and approved by the institutional committee of the Second Affiliated Hospital of Guangzhou Medical University. The patients/participants provided their written informed consent to participate in this study.

## Author Contributions

Research idea and study design: JF, YL, TQ, and LW. Data acquisition: JF, YL, LW, LC, YD, JY, XL, TQ, SL, and MH. Statistical analysis: LW, YL, and JF. Manuscript drafting: YL, TQ, and LW. Supervision: JY. All authors contributed to the article and approved the submitted version.

## Funding

This work was supported by National Key Research and Development Project (grants number: 2017YFC0113700); National Natural Science Foundation of China (grants number: 81800682; grant number: 81902928); the Medical and Health Project of Guangzhou (grants number: 20201A011079).

## Conflict of Interest

The authors declare that the research was conducted in the absence of any commercial or financial relationships that could be construed as a potential conflict of interest.
